# Safety and Tolerability of Whole Soybean Products: A Dose-Escalating Clinical Trial in Older Adults with Obesity

**DOI:** 10.3390/nu15081920

**Published:** 2023-04-16

**Authors:** Candida J. Rebello, Stephen Boué, Ronald J. Levy, Renée Puyau, Robbie A. Beyl, Frank L. Greenway, Mark L. Heiman, Jeffrey N. Keller, Charles F. Reynolds, John P. Kirwan

**Affiliations:** 1 Pennington Biomedical Research Center, Baton Rouge, LA 70808, USA; 2 United States Department of Agriculture, New Orleans, LA 70124, USA; 3 Rice Research Station, Louisiana State University Agricultural Center, Rayne, LA 70578, USA; 4 Scioto Biosciences, Indianapolis 46202, IN, USA; 5 Department of Psychiatry, University of Pittsburgh School of Medicine, Pittsburgh, PA 15261, USA

**Keywords:** soybean, older adults, obesity, tolerability, glyceollin, processing

## Abstract

Soybean products have nutrients, dietary fiber, and phytoalexins beneficial for cardiovascular and overall health. Despite their high consumption in Asian populations, their safety in Western diets is debated. We conducted a dose-escalating clinical trial of the safety and tolerability of soybean products in eight older adults (70–85 years) with obesity. Whole green soybean pods grown under controlled conditions were processed to flour (WGS) at the United States Department of Agriculture using common cooking techniques such as slicing and heat treatment. WGS incorporated into food products was consumed at 10 g, 20 g, and 30 g/day for one week at each dose. The gastrointestinal outcomes, clinical biomarkers, and adverse events were evaluated. We explored the stimulation of phytoalexin (glyceollin) production in live viable soybean seeds (LSS-G). We compared the compositions of WGS and LSS-G with commercial soybean flour and its fermented and enzymatically hydrolyzed forms. We found that although 30 g WSG was well-tolerated, and it made participants feel full. Our processing produced glyceollins (267 µg/g) in LSS-G. Processing soybean flour decreased the iron content, but reduced the oligosaccharides, which could attenuate flatulence. Providing soybean flour at <30 g/day may be prudent for overall health and to prevent the exclusion of other food groups and nutrients in older adults with obesity.

## 1. Introduction

Plants produce an array of secondary metabolites that are non-essential for basic metabolic processes, but have evolved to bestow a selective advantage against microbial or predatory attacks. These compounds are functionally defined as phytoalexins, which are synthesized de novo by the plant in response to stress, and phytoanticipins, which are pre-formed infection inhibitors. Phytoalexins are toxic to microbes but have biological activities that confer health benefits on other species [1]. Thus, resveratrol is a phytoalexin that has been widely studied for its benefits to human health [2]. Similarly, glyceollins are a group of phytoalexins synthesized de novo from the isoflavone daidzein (found in soybean) in response to environmental stresses, including fungal invasions and chemical molecules such as beta-glucan, ultraviolet rays, and metal ions. These stressors have diverse structural features so they may act simply by injuring plant cells and stimulating the phytoalexin biosynthesis pathway [3].

We investigated the concept of injuring plant cells by slicing whole green soybean pods to induce stress, and found that it stimulated the production of glyceollins when subsequently incubated [4]. Glyceollins modulate blood glucose control in animal models, which has been attributed to improvements in pancreatic beta cell function and glucose uptake in adipocytes [5,6]. We administered the glyceollin-enriched whole green soybean pods milled to flour (WGS) to mice fed an obesogenic diet and found that it reduced weight gain and fat mass [4]. Commercial soybeans are dehulled, the oil is extracted, and the remaining soybean flakes are used for food applications such as soybean flour, soybean protein concentrate, and soybean dietary fiber [7]. Unlike the soybeans used for making commercial soybean flour, glyceollin-enriched WGS was made from fresh green soybeans that were not dehulled or defatted and that contained nutrients in their original proportions. Moreover, the slicing and heat treatment used in our processing method are common cooking techniques.

Soybeans needed to be harvested at a particular reproductive stage and processed within 24 h of harvesting for glyceollin production by our processing method. Our facilities are located in Louisiana where the soybean harvesting period coincides with hurricane season, which poses many challenges for the production of glyceollin-enriched WGS. The 24-h time limitation for processing required a close proximity between the laboratory and harvesting site, which precluded obtaining fresh green soybeans from other growers. For the clinical trial reported in this paper, because of the seasonal limitations and Covid-19 restrictions from the United States Department of Agriculture (USDA), New Orleans, LA processing facility, incubation of the sliced WGS necessary for glyceollin synthesis was precluded [4].

Soybean products and consequently isoflavones form part of the traditional diets in Asian cultures. Despite their high consumption in Asian populations, some debate about their safety in the Western diet still pervades the literature [8,9,10,11]. Furthermore, including soybean in a Western diet in the amounts consumed by Asian populations may not always be culturally applicable. Moreover, processing soybeans can affect the acceptability and tolerability of their food products [12]. Therefore, our primary goal was to evaluate the safety and tolerability of food products made with WGS. To enhance the availability of glyceollin-enhanced soybean, we subsequently used our processing method to stimulate glyceollins in live viable soybean seeds that can be kept in dry storage (LSS-G), in a separate study. We report our methods and the results of the processing of LSS-G. Commercial soybean meal is processed either by fermentation or enzymatic hydrolysis to enhance the bioavailability of the isoflavones. We also present a comparison of the compositions of LSS-G, WGS, commercial soybean flour, and fermented and enzymatically hydrolyzed forms of commercial soybean flour.

## 2. Materials and Methods

### 2.1. Study Design

We conducted a three-week dose-escalating clinical trial at the Pennington Biomedical Research Center, in Baton Rouge, Louisiana. We tested the safety and tolerability of WGS incorporated into food products in older adults with obesity in preparation for an intervention trial that is ongoing in this population. The doses were determined on the basis of the dietary fiber in WGS, as there are recommendations for the consumption of dietary fiber. WGS contained approximately 36% dietary fiber. Relatively large doses of dietary fiber are tolerated if the daily intake is gradually increased and divided into small portions throughout the day [13]. The maximum recommended starting dose (MRSD) for supplemental dietary fiber was estimated at 4 g, which, when added to the average dietary fiber intake in the US of approximately 17 g [14], was not in excess of the recommended adequate intake of 25–38 g/day [15]. The dose escalation scheme recommended for first-in-human trials is rapid dose escalation initially (MRSD × 2) until the estimated desirable dose (8 g) is reached and then at a more cautious escalation (e.g., MRSD × 1.5) [16]. Participants were provided with foods containing WGS at 10 g/day (3.6 g/dietary fiber), 20 g/day (7.2 g/day dietary fiber), and 30 g/day (10.8 g/day dietary fiber) for one week at each dose, incorporated into their usual diet.

### 2.2. Participants

This clinical trial was patterned according to the National Cancer Institute’s (NCI’s) recommendation for dose escalation to determine the toxicity of a drug [17]. Accordingly, eight men and women (70–85 years) were enrolled. Participants were eligible if they presented with a body mass index between 30 and 40 kg/m^2^; no evidence of diabetes (fasting blood sugar <126 mg/dL), dementia (Mini Mental State Examination score > 25) or depression (Geriatric Depression Scale-15 [GDS-15] < 6); and if their weight was stable (<3 kg weight change in the past three months). Participants were excluded if they (a) had type 1 or type 2 diabetes; (b) had a diagnosis of cancer (within the last five years); (c) reported advanced disease of any major organs; (d) reported clinically significant gastrointestinal malabsorption syndromes, such as chronic diarrhea or celiac disease; (e) had clinically significant abnormal laboratory markers; (f) reported history of substance abuse or alcoholism or significant psychiatric disorder that would interfere with the ability to complete the study; and (g) if they were current smokers.

Screening involved measurement of the body weight, height, vital signs (blood pressure and pulse rate), complete blood count and blood chemistry-15 panel (glucose, creatinine, potassium, uric acid, albumin, calcium, magnesium, creatine phosphokinase, alanine aminotransferase, alkaline phosphatase, iron, cholesterol (total, high-density lipoprotein, and low-density lipoprotein), and triglycerides). This study was conducted according to the guidelines laid down in the Declaration of Helsinki and all procedures involving human subjects were approved by our Institutional Review Board. All participants provided written informed consent. The trial was registered with ClinicalTrials.gov Identifier: NCT04499482. The participant characteristics at screening are presented in Table 1.

### 2.3. Study Procedures

Baseline assessments included collection of a fecal sample and completion of a tolerability questionnaire. Each eligible participant completed three weekly visits to the center. Each week, participants provided a fresh stool sample, completed the tolerability questionnaire, and turned in used and unused study product packages, through which compliance was assessed. They were advanced to the next dose if they tolerated the prior dose. The study dietitian provided weekly nutrition counseling on adherence to the protocol and participants were given supplies of the study products for each week. At the end of the study, for each participant, the complete blood count and chemistry panel conducted at the screening visit were repeated. Based on our prior study in rodents showing an increase in short chain fatty acids with WGS supplementation [4], homogenized fecal samples were analyzed for short chain fatty acids, as previously described [4,18].

### 2.4. Whole Green Soybean Flour (WGS)

The soybean plants were grown under carefully monitored conditions at the Louisiana State University Agricultural Center and were harvested at reproductive stage six or when the green pods contained soybeans that filled the pods. The whole soy pods were processed at the USDA, New Orleans, LA, Food Processing and Sensory Quality Research Unit. The pods were sliced into thin cross-sections, frozen at −80 °C, lyophilized, and milled to flour. Nutrient analysis was performed at Eurofins Food Chemistry Testing (Madison, WI) using the methods of analysis from the Association of Official Analytical Chemists [19].

### 2.5. Diet Intervention

WGS was incorporated into foods and prepared in our metabolic kitchen. Each food item contained 5 g of WGS. Participants received two, four, and six food items for each day in weeks one, two, and three, respectively. A list of 14 foods developed for the study is presented in Table 2. Participants had the flexibility to consume the required number of food items for each day from among the choices provided for the week.

### 2.6. Food Tolerability

Participants completed a version of the gastrointestinal symptoms questionnaire designed as a validated assessment for dyspepsia [20], and modified it to make it specific by only including the symptoms that may be experienced with a high dietary fiber intake. The scale is ranked as none, mild, moderate, and severe for each of four gastrointestinal symptoms (bloating, abdominal rumbling, flatulence, and abdominal pain). Additionally, participants rated their stool consistency based on the Bristol Stool scale [21], as well as their stool frequency over a period of 72 h.

### 2.7. Adverse Events

Adverse events (AE) were recorded at each visit. We defined AE as any unfavorable and unintended sign (including a clinically significant abnormal laboratory finding), symptom, or disease (new or exacerbated) temporally associated with ingestion of the foods. A serious adverse event (SAE) was defined as any untoward medical occurrence that results in death, was life threatening, required inpatient hospitalization or prolongation of existing hospitalization, or resulted in persistent or significant disability/incapacity. An AE that was easily tolerated by the participant, causing minimal discomfort, and not interfering with everyday activities was classified as mild. An AE that was sufficiently discomforting to interfere with normal everyday activities was classified as moderate, and one that prevented normal everyday activities was classified as severe. Adverse events were evaluated through open-ended and non-leading verbal questioning of the participant. Based on signs, symptoms, and clinical information, a diagnosis was reached.

### 2.8. Statistical Analysis

The primary objective of the study was to determine the maximum tolerated dose of WGS. Six participants at each dose were sufficient to detect tolerability [17,22,23]. However, we enrolled eight participants to test each dose of WGS. Dose safety was investigated by compiling, by treatment (for example, 10 g dose, 20 g dose, and 30 g dose), a list of adverse events such as frequency of headaches, nausea, and vomiting. For gastrointestinal symptoms, the scale was ranked as none (no symptoms), mild, moderate, or severe intensity for each of four gastrointestinal symptoms (bloating, abdominal rumbling, flatulence, and abdominal pain). The assessment of intensity was similar to that of AEs. Moderate and severe ratings were considered as intolerance to the dose. A stool frequency of one to three/day, or three times/per week was considered regular. Therefore, a stool frequency of less than one and more than nine over a 72-h period was a consideration for determining intolerance. A stool consistency of type three to five on the Bristol Stool Scale was considered normal. Any other rating was considered as intolerance if participants also rated stool frequency and consistency as being an adverse event of a moderate to severe intensity.

According to the NCI convention for dose limiting toxicity to a drug [17]: (1) if two out of three (66%) participants were intolerant to the dose, the next lower dose was the maximum dose tolerated, and (2) if one out of three (33%) were intolerant to a dose, then that dose was considered the maximum tolerated dose. We determined that if (1) five out of eight (63%) were intolerant, the next lower dose would be adopted, and (2) if three out of eight (37%) were intolerant to a dose, then that dose would be taken as the maximum tolerated dose. Statistical tests for differences in gastrointestinal symptoms were conducted with Chi-squared tests of associations. A mixed effect linear model was used to estimate the changes in the blood biomarkers and fecal short chain fatty acids, while accounting for participant correlation across time using all of the available data from subjects tested at each dose. The outcomes were within participant changes over time. Significance was set at *p* < 0.05. All of the analyses were performed using SAS 9.4 (SAS Institute, Cary, NC, USA).

### 2.9. Study to Evaluate Nutrition and Glyceollin Content of LSS-G

To overcome the 24-h time limitation following harvesting for the processing of WGS to stimulate glyceollins, live viable soybean seeds, which are available throughout the year and are used for planting the crop, were obtained from Nutrien Ag Solutions (New Roads, LA, USA). The seeds were soaked for 30 min in peroxyacetic acid, which is a safe ingredient commonly used in the food industry [24]. The seeds were rinsed three times in water and soaked in water for 8 h. The seeds were cut for 2 min in a food processor, placed on sterile food trays, spread out in a 1.27 cm layer, and incubated for 72 h at 25 °C and 55% humidity. The cut seeds were then frozen at −80 °C, lyophilized, and milled to flour. The seeds were processed and the glyceollin content was measured by high performance liquid chromatography, at the USDA (New Orleans, LA, USA). Nutrient analysis was conducted by Eurofins (Madison, WI, USA).

## 3. Results

One participant dropped out of the clinical trial on day three of week one, stating that for personal reasons, he could not meet the demands of the study. The remaining seven participants who completed the study met the criteria for establishing tolerability at all three doses of WGS. The average compliance with the consumption of study foods was 97%, 97%, and 94% in weeks 1, 2, and 3, respectively. A summary of the gastrointestinal symptoms is presented in Table 3. There was no change in the fecal short chain fatty acids compared with baseline. There were two adverse events reported by one participant in week two as mild flatulence and one participant in week three as mild constipation. One participant had more than 1 g below normal hemoglobin levels before and after consuming the study foods. Four participants had less than 1 g below normal hemoglobin levels before starting the study and in two participants, the levels were more than 1 g at the end of the study. There were no clinically significant changes from baseline in the other blood biomarkers. There were statistically significant changes from baseline in the values for high density lipoprotein cholesterol, triglycerides, creatinine, calcium, and albumin. However, the values for each participant were within the normal range, except for one participant whose triglyceride concentrations were below normal before and after the study (Table 4). Participants reported that soybean products containing 30 g/day WGS made them feel very full.

The macronutrient and micronutrient composition and glyceollin content of WGS and LSS-G are presented in Table 5 Unlike commercial flour, WGS and LSS-G are not defatted and have a lipid content that is seven- to eight-fold higher than that of the commercially processed flours. As WGS contains the soybean pod, the dietary fiber content is ~15% higher than the LSS-G and commercial forms, largely due to increased insoluble fiber. Similar to fermentation and enzymatic hydrolysis, slicing and incubating soybean appeared to reduce its iron content by ~25%. Compared with LSS-G and the commercial forms, WSG had approximately twice the amount of calcium and 33% less phosphorus. Compared with commercial soybean flour, the enzymatically hydrolyzed and fermented soybean flours, WSG, and LSS-G had 75–100% less of the oligosaccharides raffinose and stachyose (Table 5).

## 4. Discussion

This study was conducted in preparation for an ongoing 12-week randomized controlled trial in older adults with obesity investigating the effect of whole soybean products as part of a low energy dense diet on insulin sensitivity (ClinicalTrials.gov identifier: NCT05649176). Although all doses were well-tolerated, the foods tended to make participants feel very full at the 30 g dose containing 10.8 g of dietary fiber. Our data suggest that less than 30 g/day WGS may be appropriate for older adults with obesity. Live viable soybean seeds can be stored prior to processing, which overcomes the 24-h time limitation for glyceollin stimulation in WGS. Thus, LSS-G widens the geographical availability of glyceollin-enriched soybean.

Intervention trials investigating the effect of soybean products have largely investigated the effect of soy protein supplementation on metabolic outcomes [25,26,27,28,29]. Few clinical trials have tested whole soybean products. In intervention trials, commercial soy nuts containing 10 g or 12 g dietary fiber for four weeks [30,31] or 10 g of dietary fiber for eight weeks [32] were tolerated in adults with average ages ranging from 53 to 56 years. Muffins containing approximately 10.4 g of dietary fiber from soybean flour were tolerated in adults with an average age of 55 years over a four-week intervention period [33]. The high daily intake of soy protein (37.5 g/day) and dietary fiber (13–16 g/1000 kcal) in commercially available soy foods for 24 weeks in a four-arm crossover trial led to a high drop-out rate among older adults (average age of 65 years) [34]. Together with the results from our study, the evidence suggests that 10–12 g/day dietary fiber from soybean is well tolerated.

In the present study, we did not stimulate glyceollin synthesis in WGS. Based on our prior study [4], WGS has a lower glyceollin content (175 µ/g) than LSS-G (267 µ/g). Inoculation with *Aspergillus sojae* and being incubated for 72 h produced ~1400 µg/g glyceollins, which was much higher than that produced with our method [35]. However, our processing involves the use of fundamental cooking techniques that result in food with sensory properties that conform with the natural product. Compared with commercial defatted soybean flour, WGS and LSS-G had a higher lipid content, but 66% to 80% of the lipids in soybean are unsaturated, which have a health benefit [36]. Consistent with the fermented and enzymatically hydrolyzed forms of soybean flour, the oligosaccharides raffinose and stachyose were also diminished in WSG and LSS-G. Oligosaccharides cause bloating and flatulence associated with legume consumption, and the reduction in oligosaccharides may enhance the acceptability of WSG and LSS-G [37].

The iron content diminished after processing WSG and LSS-G, similar to the commercial enzymatically hydrolyzed and fermented forms of soybean flour. The iron in soybean is non-heme iron, which is usually not absorbed as well as heme iron from animal sources, and its absorption depends on the iron status of a person [38]. The phytate content of soybean ranges from 1–1.47% on a dry matter basis, which represents 51.4–57.1% of the total phosphorous in seeds [39]. Highly charged phosphate groups make phytic acid (the salts of phytates) very reactive. If present in foods, phytic acid binds divalent cations, such as calcium, iron, zinc, and magnesium, rendering them nutritionally unavailable [40]. However, WGS had 33% less phosphorus than the commercial soybean meals. The progression from low serum iron of less than 1 g below normal before the study to more than 1 g below normal at the end of the study in two participants suggests that the satiating effect of the study foods may reduce the intake of more bioavailable sources of iron.

In a mouse model of type 2 diabetes, after the administration of fermented soybeans containing glyceollins and a mixture of isoflavones, the blood glucose response to an oral glucose challenge improved compared with mice given unfermented soy devoid of glyceollins but containing isoflavones. The effects were supported by improvements in the markers of hepatic insulin signaling [35]. Our prior studies show that in rats, glyceollins stimulate basal and insulin-stimulated glucose uptake, and experiments in 3T3-L1 adipocytes suggest that the effect may be mediated by up-regulation of the glucose transporter [5]. Furthermore, we found that in mice fed a high-fat diet supplemented with glyceollin-enhanced WGS, there was a reduction in weight gain and fat mass and increased glucose excretion [4].

The glyceollin content of the diet in the rodent studies translates into very high human doses. LSS-G contains glyceollins at approximately 267 µg/g and it would require copious quantities to be included in the diet to meet the dose for efficacy based on the rodent studies. Often, the constituents of a plant interact with one another to synergistically improve the solubility and thereby enhance the bioavailability of a particular component compared with the isolated constituent [41]. Therefore, a whole diet approach that includes LSS-G with its myriad health-promoting components in their natural proportions warrants further investigation for its effects in diseases and conditions of impaired glucose metabolism.

Similar to our tolerability study of three weeks, the main limitation of studies investigating the effects of whole soybean products is the short time frame, ranging from 4–8 weeks. Nevertheless, we found that in older adults, up to 30 g/day of WGS incorporated into food was well-tolerated. It may be prudent to consider a reduced dose so as to prevent the exclusion of other food groups and nutrients in this population accustomed to consuming a Western-type diet. Glyceollin production can be stimulated in live viable soybean seeds using common cooking methods, and its inclusion in a Western diet so as to secure the health benefits of soybeans warrants investigation.

## Figures and Tables

**Table 1:** Participant characteristics at screening.

Age ^a^	73 (2)
Body mass index (kg/m^2^) ^a^	34.0 (2.8)
Gender	
Females	3
Males	4
Race	
White	5
Black	2 ^b^

^a^ Values are mean (SD); ^b^ One male and one female.

**Table 2:** List of foods each containing 5 g of whole green soybean flour (WGS).

1	Lime pound cake
2	Banana bread
3	Corn bread
4	Spinach tomato quiche
5	Tomato soup
6	Chili with carrots and corn
7	Apple cinnamon muffin
8	Crackers
9	Herbed tomato bread
10	Berry banana shake
11	Vanilla peach shake
12	Mocha shake
13	Tropical lime shake
14	Blueberry shake

**Table 3:** Participant responses to gastrointestinal symptoms, stool consistency, and stool frequency.

Event	Baseline	Week 1	Week 2	Week 3
Bloating				
Mild	1	1	0	1
None	6	6	7	6
Total Participants	7	7	7	7
Abdominal Rumbling				
Mild	1	2	1	1
None	6	5	6	6
Total	7	7	7	7
Flatulence				
Mild	4	2	5	5
None	3	5	2	2
Total Participants	7	7	7	7
Abdominal Pain				
Mild	0	0	0	1
None	7	7	7	6
Total Participants	7	7	7	7
Stool Consistency				
1	0	0	0	1 *
3	2	2	0	0
4	3	3	5	2
5	2	2	2	4
Total	7	7	7	7
Stool Frequency				
1–2	1	2	1	1
3–4	3	4	5	3
5–6	3	1	1	3
Total Participants	7	7	7	7

* Participant rated the intensity as mild. The change from baseline for all of the measures was not statistically significant, *p* > 0.05.

**Table 4:** Changes in blood biomarkers from baseline to week 3.

Biomarker	Baseline	Week 3	*p*-Value
Chemistry-15 Panel			
Glucose (mg/dL)	94.57 ± 3.62	99.29 ± 3.62	0.28
Creatinine (mg/dL)	0.83 ± 0.08	0.91 ± 0.08	0.02 *
Potassium (mmol/L)	4.23 ± 0.15	4.28 ± 0.15	0.69
Uric acid (mg/dL)	6.06 ± 0.53	5.97 ± 0.53	0.78
Albumin (g/dL)	4.20 ± 0.09	3.96 ± 0.09	0.01 *
Calcium (mg/dL)	9.55 ± 0.10	9.21 ± 0.10	<0.01 *
Magnesium (mg/dL)	2.04 ± 0.06	2.03 ± 0.06	0.81
Creatine phosphokinase (IU/L)	186.71 ± 56.60	172.57 ± 56.60	0.49
Alanine transaminase (IU/L)	25.71 ± 3.67	26.0 ± 3.67	0.86
Alkaline phosphatase (IU/L)	72.14 ± 7.17	67.14 ± 7.17	0.19
Iron (µg/dL)	97.13 ± 9.54	84.14 ± 9.54	0.05
Total cholesterol (mg/dL)	173.57 ± 20.73	173.71 ± 20.73	0.98
High density lipoprotein cholesterol (mg/dL)	52.71 ± 3.34	48.67 ± 3.34	<0.01 *
Low density lipoprotein cholesterol (mg/dL)	99.49 ± 17.14	97.16 ± 17.14	0.66
Triglycerides (mg/dL)	106.86 ± 19.20	139.43 ± 19.20	0.04 *
Complete Blood Count			
Hemoglobin (g/dL)	13.17 ± 0.41	12.81 ± 0.41	0.20
Hematocrit (%)	39.59 ± 1.07	38.5 ± 1.07	0.16
Mean cell volume (fL)	87.46 ± 1.68	86.87 ± 1.68	0.30
Platelet count (×10^3^ cells/µL)	242.14 ± 20.11	231.86 ± 20.11	0.30
White blood cell count	6.01 ± 0.50	5.64 ± 0.50	0.15
Absolute Granulocytes (×10^3^ cells/µL)	3.34 ± 0.38	3.13 ± 0.38	0.16
Neutrophil count (×10^3^ cells/µL)	3.09 ± 0.36	2.87 ± 0.36	0.16
Eosinophil count (×10^3^ cells/µL)	0.23 ± 0.05	0.23 ± 0.05	1.00

* *p* < 0.05.

**Table 5:** Composition of the whole green soybean (WGS) and live soybean seeds (LSS-G) processed in the study and commercial soybean flour (SBF) and its fermented (FSBF) and enzymatically hydrolyzed (ESBF) forms.

Composition	WGS	LSS-G	SBF *	FSBF *	ESBF *
Macronutrients/Ash/Fiber g/100 g					
Ash	6.04	5.35	6.50	7.00	6.70
Protein	25.40	38.30	47.50	51.70	48.20
Lipid	14.00	16.60	2.20	1.90	1.50
Total carbohydrate	51.60	36.60	42.60	36.90	39.20
Dietary fiber	35.60	28.80	27.30	30.40	28.10
Insoluble fiber	32.50	23.70	-	-	-
Soluble fiber	3.10	5.05	-	-	-
Minerals mg/100 g					
Copper	0.85	0.73	0.83	0.91	0.83
Manganese	3.40	2.81	2.40	2.60	2.40
Zinc	3.38	4.49	4.00	4.50	4.00
Iron	6.17	6.78	9.10	6.70	6.00
Sodium	2.50	7.54	13.20	18.10	12.10
Calcium	549.00	284.00	260.30	286.10	267.00
Magnesium	438.00	277.00	269.70	287.60	263.00
Phosphorus	349.00	646.00	522.00	581.80	526.10
Potassium	2170.00	1880.00	2046.30	2202.60	2022.90
Oligosaccharides g/100 g					
Sucrose	1.90	5.15	4.30	Not detected	2.30
Glucose	0.22	0.12	-	-	-
Fructose	0.21	<0.10	-	-	-
Maltose	<0.10	<0.10	-	-	-
Lactose	<0.10	<0.10	-	-	-
Galactose	<0.10	<0.10	-	-	-
Raffinose	0.26	0.16	0.82	Not detected	0.43
Stachyose	0.65	0.63	2.72	Not detected	1.59
Energy kcal/100 g	297.00	340.00	-	-	-
Glyceollins µg/g	-	266.91		-	-

- indicates that data are not available; * Values are taken from [12].

## Data Availability

The datasets generated and analyzed during the current study are not publicly available, but are available from the corresponding author upon reasonable request.

## References

[1] Dixon R.A. (2001). Natural products and plant disease resistance. Nature.

[2] Guo X.F., Li J.M., Tang J., Li D. (2018). Effects of resveratrol supplementation on risk factors of non-communicable diseases: A meta-analysis of randomized controlled trials. Crit. Rev. Food Sci. Nutr..

[3] Kim H.J., Lim J.S., Kim W.K., Kim J.S. (2012). Soyabean glyceollins: Biological effects and relevance to human health. Proc. Nutr. Soc..

[4] Boue S., Fortgang I., Levy R.J., Bhatnagar D., Burow M., Fahey G., Heiman M.L. (2016). A novel gastrointestinal microbiome modulator from soy pods reduces absorption of dietary fat in mice. Obesity.

[5] Boue S.M., Isakova I.A., Burow M.E., Cao H., Bhatnagar D., Sarver J.G., Shinde K.V., Erhardt P.W., Heiman M.L. (2012). Glyceollins, soy isoflavone phytoalexins, improve oral glucose disposal by stimulating glucose uptake. J. Agric. Food Chem..

[6] Park S., Ahn I.S., Kim J.H., Lee M.R., Kim J.S., Kim H.J. (2010). Glyceollins, one of the phytoalexins derived from soybeans under fungal stress, enhance insulin sensitivity and exert insulinotropic actions. J. Agric. Food Chem..

[7] Slavin J. (1991). Nutritional benefits of soy protein and soy fiber. J. Am. Diet. Assoc..

[8] Asbaghi O., Ashtary-Larky D., Mousa A., Kelishadi M.R., Moosavian S.P. (2021). The Effects of Soy Products on Cardiovascular Risk Factors in Patients with Type 2 Diabetes: A Systematic Review and Meta-analysis of Clinical Trials. Adv. Nutr..

[9] Munro I.C., Harwood M., Hlywka J.J., Stephen A.M., Doull J., Flamm W.G., Adlercreutz H. (2003). Soy isoflavones: A safety review. Nutr. Rev..

[10] Fitzpatrick L.A. (2008). Soy isoflavones: Hope or hype?. Maturitas.

[11] Wuttke W., Jarry H., Seidlova-Wuttke D. (2007). Isoflavones—Safe food additives or dangerous drugs?. Ageing Res. Rev..

[12] Barreto N.M.B., Sandora D., Braz B.F., Santelli R.E., de Oliveira Silva F., Monteiro M., Perrone D. (2022). Biscuits Prepared with Enzymatically-Processed Soybean Meal Are Rich in Isoflavone Aglycones, Sensorially Well-Accepted and Stable during Storage for Six Months. Molecules.

[13] Grabitske H.A., Slavin J.L. (2009). Gastrointestinal effects of low-digestible carbohydrates. Crit. Rev. Food Sci. Nutr..

[14] USDA What We Eat in America, 2013–2016. https://data.nal.usda.gov/dataset/what-we-eat-america-wweia-database.

[15] National Academies Press Dietary Reference Intakes for Carbohydrate, Fiber, Fat, Fatty Acids, Cholesterol, Protein and Amino Acids (Macronutrients). http://nap.edu/10490.

[16] Foster D.M., Vicini P., Atkinson A.J., Huang S.-M., Lertora J.J.L., Markey S.P. (2012). Non-compartmental and compartmental approaches to pharmacokinetic data analysis. Principles of Clinical Pharmacology.

[17] Ivy S.P., Siu L.L., Garrett-Mayer E., Rubinstein L. (2010). Approaches to phase 1 clinical trial design focused on safety, efficiency, and selected patient populations: A report from the clinical trial design task force of the national cancer institute investigational drug steering committee. Clin. Cancer Res..

[18] Hernot D.C., Boileau T.W., Bauer L.L., Middelbos I.S., Murphy M.R., Swanson K.S., Fahey G.C. (2009). In vitro fermentation profiles, gas production rates, and microbiota modulation as affected by certain fructans, galactooligosaccharides, and polydextrose. J. Agric. Food Chem..

[19] AOAC (2019). Official Methods of Analysis of AOAC International.

[20] Bovenschen H.J., Janssen M.J., van Oijen M.G., Laheij R.J., van Rossum L.G., Jansen J.B. (2006). Evaluation of a gastrointestinal symptoms questionnaire. Dig. Dis. Sci..

[21] Lewis S.J., Heaton K.W. (1997). Stool form scale as a useful guide to intestinal transit time. Scand. J. Gastroenterol..

[22] Rebello C.J., Beyl R.A., Lertora J.L.L., Greenway F.L., Ravussin E., Ribnicky D.M., Poulev A., Kennedy B.J., Castro H.F., Campagna S.R. Safety and Pharmacokinetics of Naringenin: A Randomized, Controlled, Single Ascending Dose, Clinical Trial. Diabetes Obes. Metab..

[23] FDA Good Review Practice: Clinical Review of Investigational New Drug Applications. https://www.fda.gov/media/87621/download.

[24] United States Department of Agriculture, FSIS Environmental Safety and Health Group: Health Hazard Information Sheet: Peroxyacetic Acid (PAA). https://www.fsis.usda.gov/sites/default/files/media_file/2020-08/Peroxyacetic-Acid.pdf.

[25] Akhlaghi M., Zare M., Nouripour F. (2017). Effect of Soy and Soy Isoflavones on Obesity-Related Anthropometric Measures: A Systematic Review and Meta-analysis of Randomized Controlled Clinical Trials. Adv. Nutr..

[26] Hermansen K., Sondergaard M., Hoie L., Carstensen M., Brock B. (2001). Beneficial effects of a soy-based dietary supplement on lipid levels and cardiovascular risk markers in type 2 diabetic subjects. Diabetes Care.

[27] van Nielen M., Feskens E.J., Rietman A., Siebelink E., Mensink M. (2014). Partly replacing meat protein with soy protein alters insulin resistance and blood lipids in postmenopausal women with abdominal obesity. J. Nutr..

[28] Meyer B.J., Larkin T.A., Owen A.J., Astheimer L.B., Tapsell L.C., Howe P.R. (2004). Limited lipid-lowering effects of regular consumption of whole soybean foods. Ann. Nutr. Metab..

[29] Wiseman H., Casey K., Bowey E.A., Duffy R., Davies M., Rowland I.R., Lloyd A.S., Murray A., Thompson R., Clarke D.B. (2004). Influence of 10 wk of soy consumption on plasma concentrations and excretion of isoflavonoids and on gut microflora metabolism in healthy adults. Am. J. Clin. Nutr..

[30] Acharjee S., Zhou J.R., Elajami T.K., Welty F.K. (2015). Effect of soy nuts and equol status on blood pressure, lipids and inflammation in postmenopausal women stratified by metabolic syndrome status. Metabolism.

[31] Reverri E.J., LaSalle C.D., Franke A.A., Steinberg F.M. (2015). Soy provides modest benefits on endothelial function without affecting inflammatory biomarkers in adults at cardiometabolic risk. Mol. Nutr. Food Res..

[32] Welty F.K., Lee K.S., Lew N.S., Zhou J.R. (2007). Effect of soy nuts on blood pressure and lipid levels in hypertensive, prehypertensive, and normotensive postmenopausal women. Arch. Intern. Med..

[33] Padhi E.M., Blewett H.J., Duncan A.M., Guzman R.P., Hawke A., Seetharaman K., Tsao R., Wolever T.M., Ramdath D.D. (2015). Whole Soy Flour Incorporated into a Muffin and Consumed at 2 Doses of Soy Protein Does Not Lower LDL Cholesterol in a Randomized, Double-Blind Controlled Trial of Hypercholesterolemic Adults. J. Nutr..

[34] Matthan N.R., Jalbert S.M., Ausman L.M., Kuvin J.T., Karas R.H., Lichtenstein A.H. (2007). Effect of soy protein from differently processed products on cardiovascular disease risk factors and vascular endothelial function in hypercholesterolemic subjects. Am. J. Clin. Nutr..

[35] Park S., Kim D.S., Kim J.H., Kim J.S., Kim H.J. (2012). Glyceollin-containing fermented soybeans improve glucose homeostasis in diabetic mice. Nutrition.

[36] USDA Food Data Central, Search Results Soybeans, Green, Raw. https://fdc.nal.usda.gov/fdc-app.html#/food-details/169282/nutrients.

[37] Rebello C.J., Greenway F.L., Finley J.W. (2014). A review of the nutritional value of legumes and their effects on obesity and its related co-morbidities. Obes. Rev..

[38] Hurrell R., Egli I. (2010). Iron bioavailability and dietary reference values. Am. J. Clin. Nutr..

[39] Guo M., Guo M. (2009). Chapter 7—Soy Food Products and Their Health Benefits. Functional Foods.

[40] Arntfield S.D., Yada R.Y. (2018). 7—Proteins from oil-producing plants. Proteins in Food Processing.

[41] Wagner H., Ulrich-Merzenich G. (2009). Synergy research: Approaching a new generation of phytopharmaceuticals. Phytomedicine.

